# The EVENDOL Pain Scale Validation for Acute Non‐Procedural Neonatal Pain in Term Neonates: Reliability and Validity in Maternity Wards

**DOI:** 10.1002/pne2.70008

**Published:** 2025-06-06

**Authors:** Lucie Calamy, Elisabeth Fournier‐Charrière, Patricia Martret, Patricia Cimerman, Claire Boithias, Thierry Debillon, Ricardo Carbajal, Bruno Falissard, Elizabeth Walter‐Nicolet

**Affiliations:** ^1^ Medicine and Neonatal Intensive Care Unit Saint Joseph Hospital Paris France; ^2^ Paediatric Pain Centre Trousseau Hospital, Assistance Publique – Hôpitaux de Paris Paris France; ^3^ Pediatric and Neonatal Intensive Care Unit, Bicêtre Hospital, Assistance Publique – Hôpitaux de Paris Le Kremlin‐Bicêtre France; ^4^ Neonatal Intensive Care Unit University Hospital of Grenoble Alpes Grenoble France; ^5^ Paediatric Emergency Department Trousseau Hospital, Assistance Publique – Hôpitaux de Paris Paris France; ^6^ INSERM, U1153, Epidemiology and Statistics Sorbonne Paris Cité Research Center Obstetrical, Perinatal and Pediatric Epidemiology Team Paris France; ^7^ AP‐HP, INSERM U1178, University Paris‐Sud, University Paris‐Descartes Paris France

**Keywords:** acute non‐procedural pain, EVENDOL, maternity wards, term neonates

## Abstract

The assessment of acute non‐procedural pain in term neonates in maternity wards is challenging due to the difficulty in selecting an appropriate scale and the time‐consuming nature of the process. This can lead to inadequate neonatal pain management. To validate the EValuation ENfant DOuLeur (EVENDOL) pain scale for acute non‐procedural pain in term neonates in maternity units by comparing it with the Echelle Douleur et Inconfort du Nouveau‐né (EDIN) used as a reference. We hypothesized that EVENDOL would be equivalent to EDIN in assessing acute non‐procedural neonatal pain, with better appearance. Prospective multicentric non‐interventional open study. Term neonates over 37 weeks' gestation in the delivery room and postnatal care units, with or without acute non‐procedural pain, before and after analgesia. Cronbach's α coefficient, intraclass correlation (ICC), and correlation between EVENDOL and EDIN scores, documented by the researchers and the caregivers at rest and mobilization, before and after oral paracetamol, were measured. Ninety‐one neonates were included: 48 (51%) had pain and 43 (47%) had no pain. Before analgesia, the Cronbach coefficient was above 0.80, the ICC (25th–75th interquartile ranges [IQ]) were 0.84 (0.77–0.89) and 0.90 (0.85–0.93) at rest and mobilization, respectively. Seventeen patients received oral acetaminophen and were re‐assessed. Psychometric values remained good after analgesia (Cronbach coefficient above 0.80, ICC [IQ]: 0.65 [0.26–0.85] and 0.76 [0.45–0.91]) at rest and mobilization, respectively. The feasibility and ease of use were better for EVENDOL for researchers and caregivers. EVENDOL is suitable for the assessment of acute non‐procedural neonatal pain for term neonates in the maternity wards.

**Trial Registration:** ClinicalTrials.gov identifier: NCT02819076, registered in June 2016 as EVENDOL scale validation for at term newborn

## 1 Introduction

Objective and reliable assessment is the first step in good quality pain management, but choosing the right tool can be difficult in neonates, especially in maternity wards, delivery room (DR), and postnatal care, where turnover is high and stress can be intense. A survey conducted in 2014 in the Paris region, France, revealed that 68% of the 96 maternity wards assessed acute or prolonged pain in term neonates [1]. The remaining 32% of maternity wards did not assess neonatal pain due to time‐consuming and inadequate scales for these patients, choosing to follow their own clinical observations [1]. A Spanish study showed that the grading of procedural pain in the DR or postnatal unit was subjective, with a pain scale used in only 12.5% of maternity wards [2]. Finally, a monocentric study conducted in a French level‐III delivery room showed that a systematic assessment led to a fourfold relative increase in the pain diagnosis rate [3].

There are many scales for assessing procedural pain in neonates but fewer for acute non‐procedural or prolonged pain, and most of the studies have been conducted in Neonatal Intensive Care Units (NICUs) [4]. The Echelle Douleur et Inconfort du Nouveau‐né (EDIN) is a behavioral pain scale widely used not only in the NICUs but also in maternity wards for term neonates, although it is not suitable for this population. Indeed, it has been validated to assess prolonged pain in preterm neonates in the NICUs, after an observation time of several hours (8 h in the validation study), both outside and during care [5]. The EDIN score at a given time reflects the presence of comfort or pain in the preceding hours [5]. Other scales than EDIN, such as the COMFORT‐neo [6], can be used in term neonates in the NICUs, especially in ventilated or sedated neonates, but there is a lack of tools to assess acute or persistent non‐procedural pain in term neonates in the maternity wards. In the 2014 French survey [1], the EDIN scale was used in 80% of assessments in the maternity wards, even though 40% of nurses considered it inappropriate for use with neonates in the DR or postnatal wards [1].

EVENDOL (EValuation ENfant DOuLeur) is a behavioral five‐item scale validated for acute or persistent pain in the accident and emergency departments (AEDs) for children under 7 years of age [7]. It was drawn up and validated to allow a quick and easy pain assessment in a place where turnover and stress can be high, and where children with pain have to be quickly identified and treated [7]. In the initial EVENDOL validation study, only 28 neonates were included [7]. The psychometric properties of EVENDOL are comparable to those of EDIN; both are behavioral scales used for young children. EVENDOL seemed to us to be a more appropriate tool than EDIN to assess acute non‐procedural or persistent pain for term neonates in the maternity wards (DR and postnatal unit). To this end, we aimed to validate EVENDOL in a prospective cohort of term neonates with or without pain in the maternity ward, by comparing it with EDIN, the usual tool in these French units [1].

## 2 Methods

### 2.1 Design

Prospective, multicentric, non‐interventional, open study conducted in four maternity wards in the region of Paris, France.

Our main hypotheses were that EVENDOL in term neonates:

- Has a good internal consistency,
- Has a good inter‐rater reliability,
- Has a good correlation with EDIN scores,
- Has a good construct validity
  ○ Contrasting scores between neonates with pain and without pain
  ○ Sensitivity to change (the score decrease after analgesic treatment)
  ○ Correlation with an attributed numeric rating scale (NRS) between 0 and 10 by the researcher and the caregiver.

Our secondary hypotheses were that EVENDOL in term neonates:

- As a good appearance validity (feasibility and ease of use)
  ○ Is preferred by 70% of caregivers over EDIN
  ○ Has easier items to score than EDIN
  ○ Is faster to score than EDIN
- Has a discriminant validity with other variables such as hunger (time between last meal and evaluation).

### 2.2 Main Outcome Measures

- EVENDOL's internal consistency given by Cronbach's alpha (*α*) coefficient (very good if > 0.8) [8],
- EVENDOL's interrater reliability by correlating researcher's and caregivers' scores at the two time points (rest and mobilization) and calculating the intra‐class correlation (ICC), good if > 0.7 [9, 10].
- High correlation (more than 0.7) between EVENDOL and EDIN scores, measured by a Spearman correlation,
- Higher EVENDOL scores in neonates with pain than in neonates without pain, lower EVENDOL scores after analgesia, and correlation between EVENDOL and attributed NRS.

### 2.3 Secondary Outcomes

- Overall rater satisfaction at the end of the scoring (“which of the two scales do you prefer”?, “which item, if any, is difficult to apply?” and “which scale do you think is most appropriate for this patient?”), and time taken to complete the assessment for both scales,
- EVENDOL discriminates with hunger.

### 2.4 Inclusion Criteria

– Term neonates (≥ 37 weeks' gestational age [GA]) in the maternity unit, suspected of being in pain or not.

### 2.5 Non‐Inclusion Criteria

– Major congenital abnormalities
– Parents' inability to speak French
– Absence of a referent researcher for the study
– Neonatal palliative care

### 2.6 Setting

The study took place over two separate periods:

– From March 2014 to January 2016, which was the pilot study (unpublished data),
– From January to June 2019, after the recruitment of a research nurse.

Three researchers supervised the study, two pediatricians and a research nurse. Enrolment and assessments were always done by the same pediatrician and research nurse; the second pediatrician did the analysis and was blinded to all interventions.

### 2.7 Pain Evaluation

The EDIN was used daily and routinely by all nurses at all four sites prior to the study and was the reference scale for pain diagnosis and management, with a treatment threshold of 5 out of 15 [5]. The EDIN has five items (facial activity, body movements, quality of sleep, quality of contact with nurses, and consolability) (Figure S1). In NICUs, the prolonged observation time required to score the EDIN includes mobilization and/or examination, as this scale needs to be assessed before, during, and after patient care [5]. As it was not possible to score the EDIN after several hours of observation in the maternity ward, it was scored after observation of the neonate at rest and on mobilization during examination in the DR or postpartum ward, depending on where it was assessed. The EDIN is usually printed on a black and white A4 sheet.

EVENDOL is also a five‐item behavioral scale (facial expression, movements, vocal or verbal expression, postures, and interaction with the environment) (Figure S2), with a treatment threshold of 4 out of 15 [7]. EVENDOL was rated at rest and during examination, as required for this scale [7]. To ensure good interrater reliability within the caregivers, all midwives, nurses, and pediatric nurse assistants in each center were trained in the use of EVENDOL by two researchers prior to the study (verbal and video training sessions with the DVD created by the team who first validated EVENDOL [7]). The usual presentation of EVENDOL is a colored scale, purple, white, and beige, in a pocket ruler (size 10 × 15 cm). This presentation was not shown to the learners' caregivers in order not to influence their opinion of the scale's appearance and ease of use, and to avoid any cognitive bias. As with the EDIN, the EVENDOL scale was printed on an A4 black‐and‐white sheet.

Following the birth, the caregiver was responsible for contacting the researcher if present to assess the neonates in the DR or postpartum ward. Neonates without pain were selected at random, following parental consent for inclusion in the study. Neonates exhibiting signs of pain (clinical signs) or risk factors of pain (e.g., instrumental extraction) were assessed after the caregiver contacted the researcher to include the patient. All neonates were assessed using the two scales, EDIN and EVENDOL, both at rest and during mobilization and examination, regardless of where the neonate was (DR or postnatal ward), before any analgesia (T1). The order of the scales was randomized for each research file to avoid raters getting used to a particular order. Each enrolled neonate was assessed simultaneously by a researcher and a caregiver, at rest and during mobilization, using both scales. A timer was started at the start of the assessment and stopped when the assessment was completed by both raters. Finally, the researcher and the caregiver also had to give an attributed numeric rating scale (NRS) between 0 and 10 for each patient.

If pain was diagnosed (total EDIN score ≥ 5), a treatment with oral paracetamol (15 mg/kg) could be administered according to each hospital's practice, and a second assessment had to be performed 1–2 h later by the same researcher/caregiver pair (T2). Between the two assessments, neonates were managed according to the practices of each centre and the neonatal condition (skin‐to‐skin contact, breastfeeding, observation). For the second observation period, the same procedure was used with the same researcher and caregiver.

We defined three pain groups: no pain (EDIN < 5 for all measures [researcher and nurse, at rest and mobilization]), mild‐to‐moderate pain (5 ≤ EDIN < 10 for at least one measure), and severe pain (EDIN ≥ 10 for at least one measure).

### 2.8 Statistical Analysis

Descriptive analyses are presented as medians, 25th–75th interquartile ranges (IQ). Cronbach's *α* coefficient and ICC are presented with their 95% confidence interval (CI). The ICC was calculated by randomly analyzing the scale, completed by one of the observers, and then comparing it with the data reported by the other observer. Correlations between scores were reported using the Spearman test (non‐parametric data) [11]. EDIN and EVENDOL are numerical, continuous, unidimensional scales. To study the construct validity and to compare children with and without pain, we needed about 30 children for each group (no pain, mild‐to‐moderate pain, severe pain). This allows for the comparison between groups a power of 80% to highlight a difference corresponding to an effect size of 0.75. Data from the two periods were pooled. The α‐risk was 5% for all tests, and *p* < 0.05 was considered significant. Analyses were performed with the R software, version 3.6.1. The COSMIN Study Design checklist for patient‐reported outcome measurement instruments was used.

### 2.9 Ethics

The study protocol was approved by the Ethics Committee of the Paris Saint Joseph Hospital (Groupe Ethique Recherche Médicale) and the Committee for the Protection of People Participating in Biomedical Research, Ile‐de France Region (IRB registration #0001072), France, in June 2014. As this study was non‐interventional, in accordance with French law, verbal and written information was provided to one or both parents, who had to give their verbal consent, which was recorded in the neonate's medical record. In case of refusal, the neonate was not included in the study and was managed according to the local protocol. For each neonate, a researcher explained the study orally and in writing to one or both parents and obtained their verbal consent.

## 3 Results

### 3.1 Patients' Characteristics

From March 2014 to June 2019, 91 patients (34 in the first period, 57 in the second period) were included in the four hospitals (A: 57 neonates, B: 17, C: 7, D: 10). There were 51 (56%) boys. The median (IQ range) gestational age, birth weight (grams [g]), and 5‐min Apgar score were 39 GW (39–40.5), 3340 g (3015–3610) and 10 (10) respectively. All but four neonates (4.4%) were born by cephalic presentation. There were 55 (60.5%) non‐instrumental vaginal deliveries, 22 (24.2%) instrumental vaginal deliveries, and 14 (15.4%) cesarean sections.

### 3.2 Pain Evaluation

**FIGURE 1:**
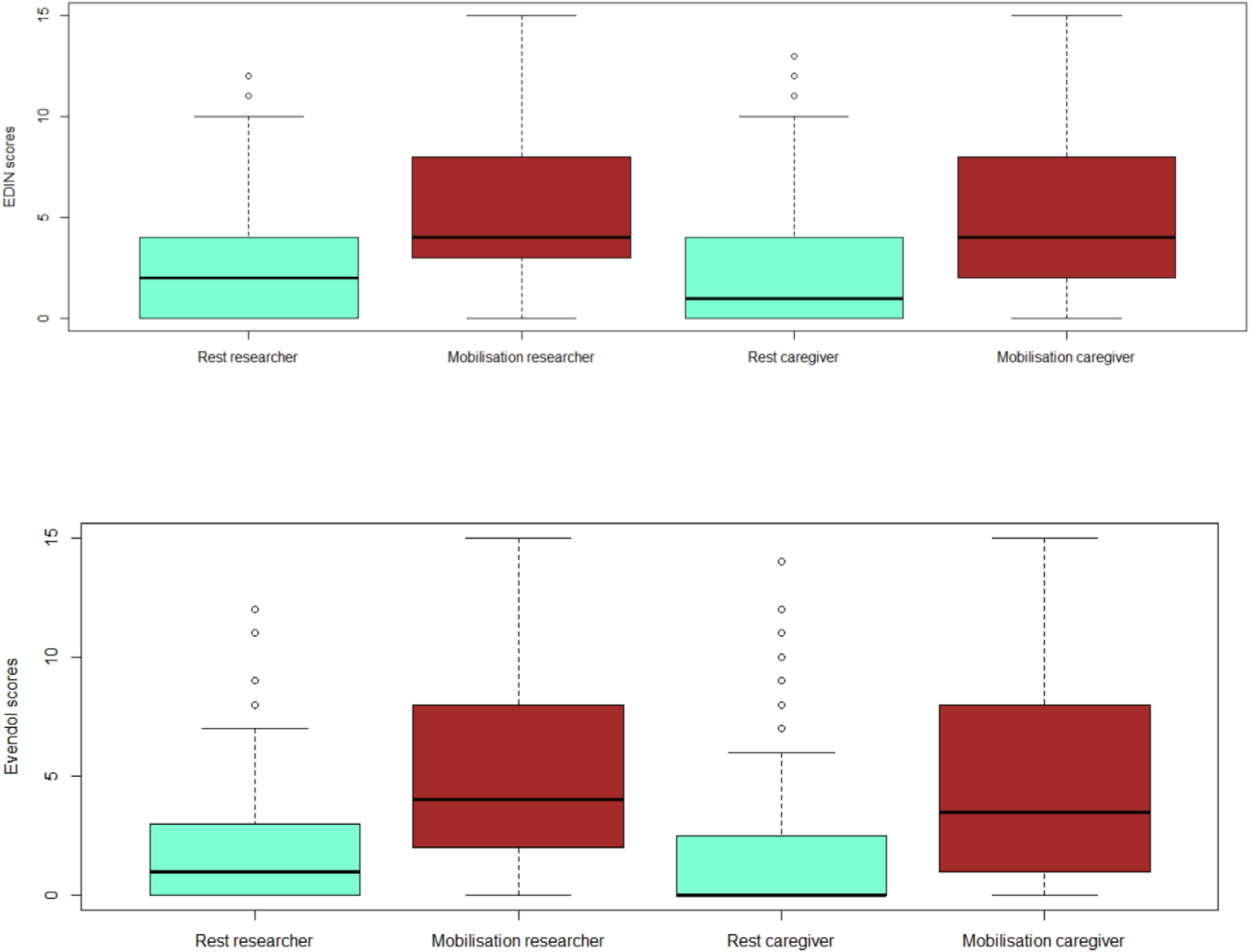
Distribution of EDIN and EVENDOL pain score at rest and mobilization with researcher and caregiver before analgesia in the 91 patients.

**FIGURE 2:**
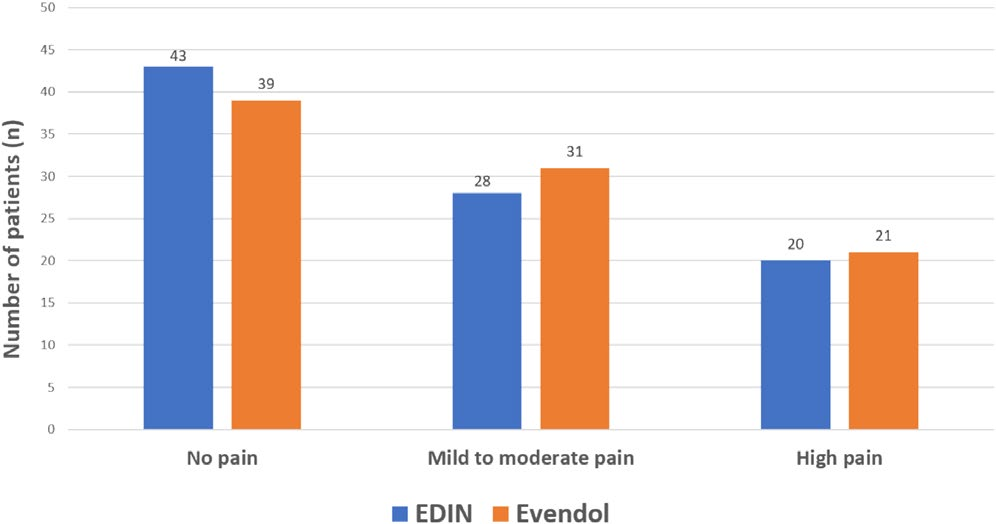
Distribution of neonatal pain scores according to the three defined groups and the EDIN and EVENDOL scales. No pain (EDIN < 5 for all measures (researcher and nurse, at rest and mobilization)), mild to moderate pain (5 ≤ EDIN < 10 for at least one measure), high pain (EDIN ≥ 10 for at least one measure).

All neonates were assessed in the DR or postnatal ward during the first 48 h (h) of life. According to our classification, 43 (47%) patients had no pain, 28 (31%) patients had mild to moderate pain, and 20 (22%) patients had severe pain. Perinatal trauma was noted in 49 (54%) of the patients. The distribution of pain scores for both scales at first assessment (T1), at rest and at mobilization for the 91 patients, is shown in Figure 1. Figure 2 shows the distribution of neonates, according to the three defined groups, and evaluation with EDIN and EVENDOL scales.

### 3.3 Internal Consistency and Interrater Reliability

Cronbach's coefficient ranged from 0.80 to 0.9 at T1 and T2 (Table 1). Interrater reliability, as measured by ICC, was 0.84 (0.77–0.89) at rest and 0.90 (0.85–0.93) at mobilization at T1. At T2 (after analgesia), the ICC between the researcher and nurse remained good but was lower than at T1 (0.65 [0.26–0.85] and 0.76 [0.45–0.91] at rest and mobilization, respectively).

**TABLE 1:** Cronbach coefficient at rest and mobilization for researcher and caregiver before and after analgesia.

EVENDOL	Cronbach (CI)
Rest T1	
R (*n* = 90)	0.82 (0.71–0.90)
C (*n* = 91)	0.91 (0.86–0.95)
Mobilization T1	
R (*n* = 90)	0.91 (0.88–0.93)
C (*n* = 91)	0.91 (0.87–0.93)
Rest T2	
R (*n* = 17)	0.88 (0.55–0.98)
C (*n* = 17)	0.87 (0.54–0.98)
Mobilization T2	
R (*n* = 17)	0.91 (0.47–0.92)
C (*n* = 17)	0.91 (0.80–0.95)

Abbreviations: C, caregiver; R, researcher; T1, before analgesia; T2, after analgesia.

### 3.4 Correlation Between EDIN and EVENDOL Scores

The Spearman correlation, comparing EVENDOL and EDIN scores, at rest and mobilization, for researcher and carer was good: above 0.85 at T1, and between 0.65 and 0.91 at T2 (Table 2).

**TABLE 2:** Spearman coefficient between EVENDOL and EDIN scores at rest and mobilization for researcher and caregiver before and after analgesia.

Comparative EVENDOL	*ρ* coefficient	*p*
Rest T1		
EDIN–R (*n* = 90)	0.85	< 10^−3^
EDIN–C (*n* = 91)	0.88	< 10^−3^
Mobilization T1		
EDIN–R (*n* = 90)	0.93	< 10^−3^
EDIN–C (*n* = 91)	0.95	< 10^−3^
Rest T2		
EDIN–R (*n* = 17)	0.65	0.002
EDIN–C (*n* = 17)	0.84	< 10^−3^
Mobilization T2		
EDIN–R (*n* = 17)	0.67	0.003
EDIN–C (*n* = 17)	0.91	< 10^−3^

Abbreviations: C, caregiver; R, researcher; T1, before analgesia; T2, after analgesia.

### 3.5 Construct Validity

**FIGURE 3:**
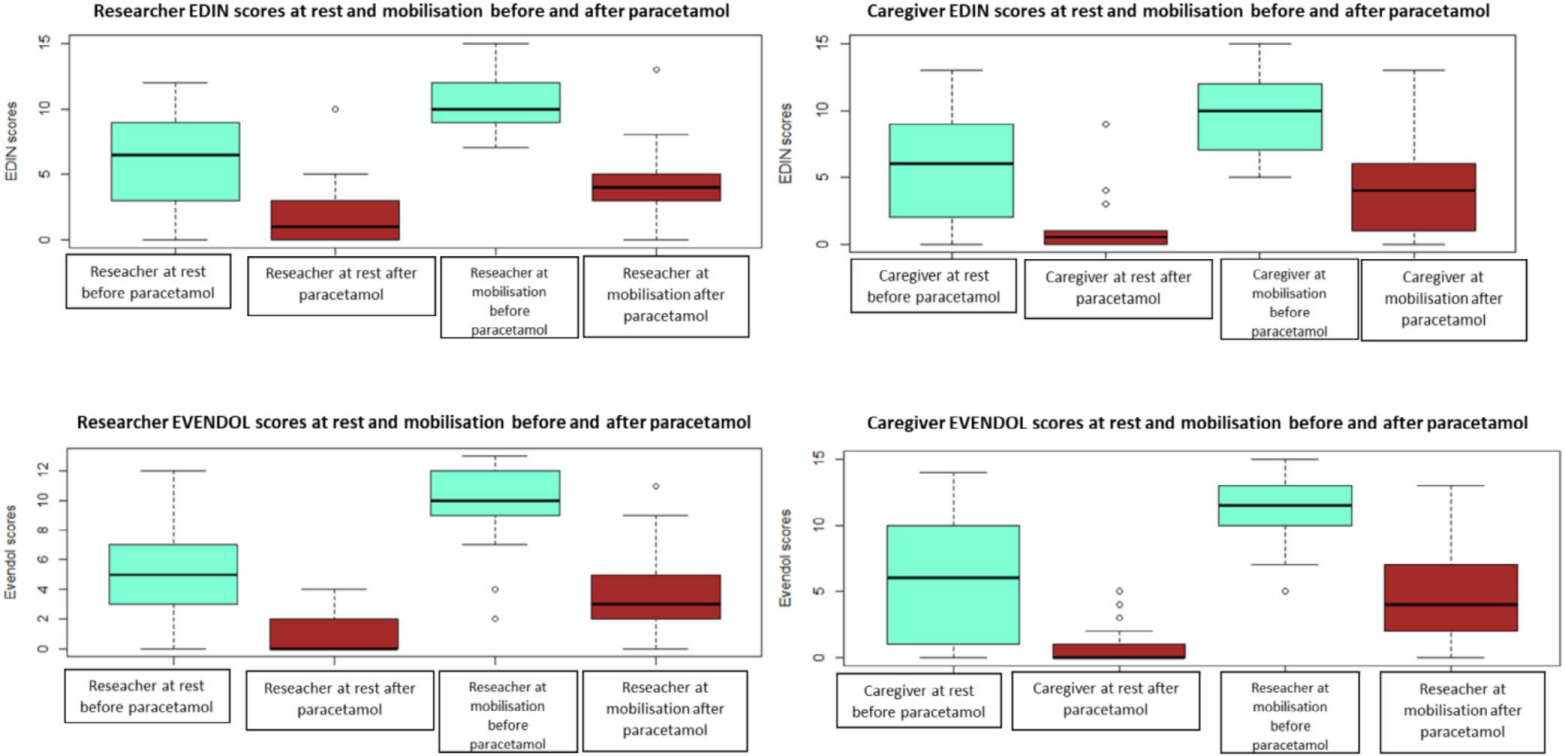
Distribution of EDIN and EVENDOL pain score at rest and mobilization for researcher and caregiver before and after analgesia in the 17 patients who received oral paracetamol.

– Neonates considered a priori non‐painful, and randomly selected mostly in the postpartum ward, had a mean EDIN and EVENDOL scores of 0 at rest, and of 1.8 and 1.4 at mobilization, respectively.
– The mean EDIN and EVENDOL scores in the 48 painful patients were 5 and 4, and 9.1 and 9.4 at rest and mobilization, respectively. Of these 48 painful patients, 17 (35%) received paracetamol and had a second assessment. The other 31 were treated with non‐pharmacological measures, mainly skin‐to‐skin contact with the mother and/or breastfeeding. These neonates were closely monitored but not re‐evaluated, as specified in the study protocol. The 17 patients were reassessed between 45 and 90 min after paracetamol administration: the mean EDIN and EVENDOL scores were 1.56 and 0.92 at rest, and 4.1 and 4.15 at mobilization, respectively. The distribution of pain scores for both scales after paracetamol, at rest and at mobilization for the 17 patients, is shown in Figure 3.
– The EVENDOL scores were compared with the NRS scores provided by the researcher and the caregiver: the correlations ranged from 0.78 to 0.93 depending on the time point (Table S1).

### 3.6 Overall Satisfaction, Facility of Use and Time to Achieve the Scales

EVENDOL was the preferred scale for 59/89 (66%) of the 91 ratings, and the most appropriate scale for 66/89 (74%) of the ratings by both caregivers and researchers. Two items were difficult to rate on the EDIN: “sleep” was mentioned by 24 (26%) patients and “quality of contact with nurse” by 3 (3%). EDIN was also reported to be time‐consuming and difficult to use in DR by 5 (5.5%) caregivers. For EVENDOL, “Position” and “Relationship with environment” caused some difficulties for 7 (8%) and 4 (4.5%) patients, respectively.

The time taken to complete the assessment was comparable for both scales (Table 3). The researchers rated faster than the caregivers, especially at T1 (EVENDOL T1: 41 s vs. 61 s, *p* < 10–3; EDIN T1: 50s vs. 60s, *p* = 0.06. EVENDOL T2: 38 s vs. 50s, *p* = 0.06; EDIN T2: 43 s vs. 50s, *p* = 0.60).

**TABLE 3:** Time (seconds) to achieve EDIN and EVENDOL before and after analgesia for researcher and caregiver.

	EDIN–R	EVENDOL–R	*p*	EDIN–C	EVENDOL–C	*p*
T1‐Mean (SD)	50.5 (31.6)	41 (23.4)	0.02	59.9 (34)	61 (47)	0.83
T2‐Mean (SD)	43.3 (40.5)	38 (15)	0.65	50.3 (23.6)	49.7 (16.5)	0.95

Abbreviations: C, caregiver; R, researcher; T1, before analgesia; T2, after analgesia.

The missing data for hunger were too large to allow us to perform the analyses. Therefore, we could not verify that there was no correlation between hunger and the EVENDOL score.

## 4 Discussion

Our results show that EVENDOL for term neonates in the maternity ward meets the criteria for reliable assessment of acute non procedural pain in this population: very good internal consistency and inter‐rater reliability, good construct validity and very good correlation with EDIN, the scale choosen as a reference. I also has a very good correlation with the NRS, and a better feasibility and ease of use than EDIN. The validation of EVENDOL responded to an important clinical need in the maternity ward for term neonates: a tool that is simple, easy to understand and use, for all types of pain (acute non‐procedural and prolonged).

A suitable and reliable tool is needed to easily detect and assess pain in term neonates in the DR where patient turnover is high, as well as in the postpartum ward where caregivers cannot allow a very long time to each neonate. Pain in the maternity ward is probably under‐evaluated, and subsequently under‐treated, as in the NICUs [12]. for term neonates in the maternity [13]. Well‐being term neonates, even if in pain, are obviously different than ventilated or sedated neonates (preterm or not), and the scales used in the NICUs such as EDIN [5] or COMFORT‐neo [6] are not suitable for these patients. Initially approved for use in emergency departments, EVENDOL quickly became popular in France for the assessment of children under 7 years of age and is now used in most French AEDs, general pediatric wards and out‐of‐hospital emergency medical teams [14]. It has been translated into English [15], Portuguese [16], Dutch [15], Spanish, German, and several European countries claim its usefulness and use it in their own languages. A systematic review mentioned EVENDOL as a suitable scale with a lower risk of bias [13]. Its items seemed to us to be appropriate for term neonates in the maternity ward, and many caregivers expected validation for use in the early neonatal period, expressing a real need. Children of all ages were included in the initial validation, except very young babies and neonates who were under‐represented, needing further studies [7]. However, in the EVENDOL prehospital study, 150 neonates requiring transfer were included [14]. Although validated specifically for preterm neonates in the NICU [5], EDIN is the most widely used pain scale in French maternity units [1], demonstrating a deficit in this setting, but also justifying the choice of this behavioral scale as a reference to compare to EVENDOL. EDIN, which is widely used in several countries for persistent pain [2, 4, 17, 18, 19], was also selected for its concurrent validity [20]. Nevertheless, there are several arguments for not using it on term neonates in maternity wards: the long time required for observation by the same nurse, the sleep observation and the quality of contact with the nurse [1, 5]. Indeed, sleep is erratic in the first days of life. In addition, unlike in the NICUs, in the maternity ward, the caregivers are not always present with the neonate to assess him or her, which makes it difficult to carry out prolonged observation as recommended for the EDIN [5]. In our study we compared the time to reach EDIN and EVENDOL and found almost the same time for both scales. However, these results are misinterpreted because EDIN should have been administered after a longer period of observation than the few minutes at the neonate's side, and was not used appropriately. On the other hand, EVENDOL was designed to be administered in a few minutes [7, 14]. Our study showed a very good correlation (0.85) between the two scales indicating that EVENDOL has a very good construct validity. In the initial validation study and in the study for out‐of‐hospital emergency transport, EVENDOL also showed a good correlation (0.75) with the Numeric Rating Scale [14]. We also showed a good correlation with the attributed NRS, confirming a good construct validity.

EVENDOL items mainly focus on clinical features of the patient that are present in most neonatal pain scales (crying, facial expression, and body movements) and are not subjective. Crying is not an EDIN item, which is not surprising as validated for prolonged pain in preterm neonates who are weaker than term neonates to cry or scream heavily, or who were intubated. We assume than in term neonates with acute non procedural pain, cry will be a major clinical sign. In case of doubt (e.g., painful clinical situation and absence of crying), psychomotor atonia should be considered, which can also be diagnosed with EVENDOL. Mobilization or examination may cause a cry, with the restriction, of course, that the child must not be mobilized if the pain is obvious (e.g., bone fracture). As other pain scales, EVENDOL is only a tool, and you should not hesitate to ask the parents or another caregiver for their opinion in order to best assess the pain and adapt the treatment. Similarly, mobilization is not mentioned in the initial validation of EDIN [5]. However, prolonged observation during and after care obviously involves manipulation of preterm neonates, which can cause pain. Therefore, we asked researchers and caregivers to mobilize the newborns for this study also to evaluate the EDIN. Mobilization revealed pain in several cases in our study. “Position” and “relationship to environment,” two of the EVENDOL items, were reported as difficult by caregivers for only 7 and 4 patients, respectively. The item “relationship to environment” is comparable to the two EDIN items that are also difficult to assess, “quality of contact with nurse” and “consolability.” This demonstrates the difficulty of assessing pain in a neonate and the need to first observe and try to establish a relationship with him/her.

EVENDOL was the preferred scale in this study. Although it has not been shown to protect caregivers from cognitive bias, the availability of a pocket ruler size scale also supports its use in the maternity unit, as it may allow for quicker bedside pain assessment. We could not confirm with this study that EVENDOL discriminates with other situations such as hunger, but the previous studies on EVENDOL showed that it was able to discriminate pain from anxiety and was not affected by fever, fatigue or hunger [7, 14].

This study has several limitations: First, the recruitment period was longer than expected due to the lack of availability of the researcher during the first period. During this time, the assessment practices for neonatal pain management in our centers did not change. A second EVENDOL training course was completely redone by the research nurse for the teams with the training DVD. However, the inter‐rater reliability between caregivers for EVENDOL was not assessed before the study. We expected it to be good, as in previous studies [7, 14]. Furthermore, the inter‐rater reliability between researchers and caregivers (ICC) was excellent, suggesting a good appropriateness of the tool. Second, we did not recruit as many neonates with pain as we had expected. This may reflect the fact that severe pain is a rare event in maternity units, and/or that neonates with severe pain are treated in the most appropriate unit (NICU, surgery, etc.). Also, only 17 neonates with pain were treated with paracetamol and reassessed. Despite this, the validity criteria for EVENDOL remain good, particularly Cronbach's coefficient, inter‐rater reliability and construct validity, leading to confidence in the validity of the scale in maternity wards, including both painful and non‐painful neonates.

## 5 Conclusion

Pain in the maternity ward is difficult to assess without appropriate tools and is likely to be under‐diagnosed, leading to inadequate pain management in term neonates. Our study is the first to validate a suitable scale that has already been tested and used worldwide in different clinical settings, translated into several languages and meets a real need. Therefore, EVENDOL could be a very good scale to assess acute non‐procedural pain from birth to the age of self‐assessment in many settings, except for ventilated or sedated patients in the NICU, for whom most appropriate tools need to be chosen. Further studies should confirm this after EVENDOL has been widely used in maternity units.

## Author Contributions

Elizabeth Walter‐Nicolet contributed to the study conception and design, which was validated by Bruno Falissard, Ricardo Carbajal, and Elisabeth Fournier‐Charrière. Material preparation and data collection were performed by Lucie Calamy, Patricia Cimerman, and Patricia Martret. Funding acquisition was done by Elizabeth Walter‐Nicolet. Supervision of data collection in the hospitals were performed by Elizabeth Walter‐Nicolet and Claire Boithias. Analysis was performed by Lucie Calamy and Elizabeth Walter‐Nicolet. The first draft of the manuscript was written by Elizabeth Walter‐Nicolet. All authors commented on previous versions of the manuscript. All authors read and approved the final manuscript. Submission was done by Elizabeth Walter‐Nicolet.

## Ethics Statement

The study protocol was approved by the local ethics committee and by the Committee for the Protection of People Participating in Biomedical Research, Ile‐de France Region, France. As this study was non‐interventional, according to French law, oral and written information was given to one or both parents who had to give their oral non‐opposition, which was recorded in the neonate's medical file.

## Consent

Consent to Participate: An oral and written information was given to one or both parents who had to give their oral non‐opposition, which was recorded in the neonate's medical file. In case of refusal, the neonate was not included in the study and managed according to local protocol. For each neonate, a researcher explained the study orally and with a written letter to one or both parents and collected their oral non‐opposition.

Consent to Publish: All parents who consented their child to be involved in the study were aware in the information letter of the aims of the research and the later publication of its results in a scientific review. All data are pooled and strictly anonymous. We had no opposition.

## Conflicts of Interest

The authors declare no conflicts of interest.

## Supporting information


Data S1.

## Data Availability

The data that support the findings of this study are available from the corresponding author upon reasonable request.
