# Supplementary material for: The EVENDOL Pain Scale Validation for Acute Non‐Procedural Neonatal Pain in Term Neonates: Reliability and Validity in Maternity Wards
Source: Paediatr Neonatal Pain. 2025 Jun 6;7(2):e70008. doi: 10.1002/pne2.70008 (PMC12143344; doi:10.1002/pne2.70008)
Supplement: Supplementary file 1 — Data S1. [file PNE2-7-e70008-s001.docx]

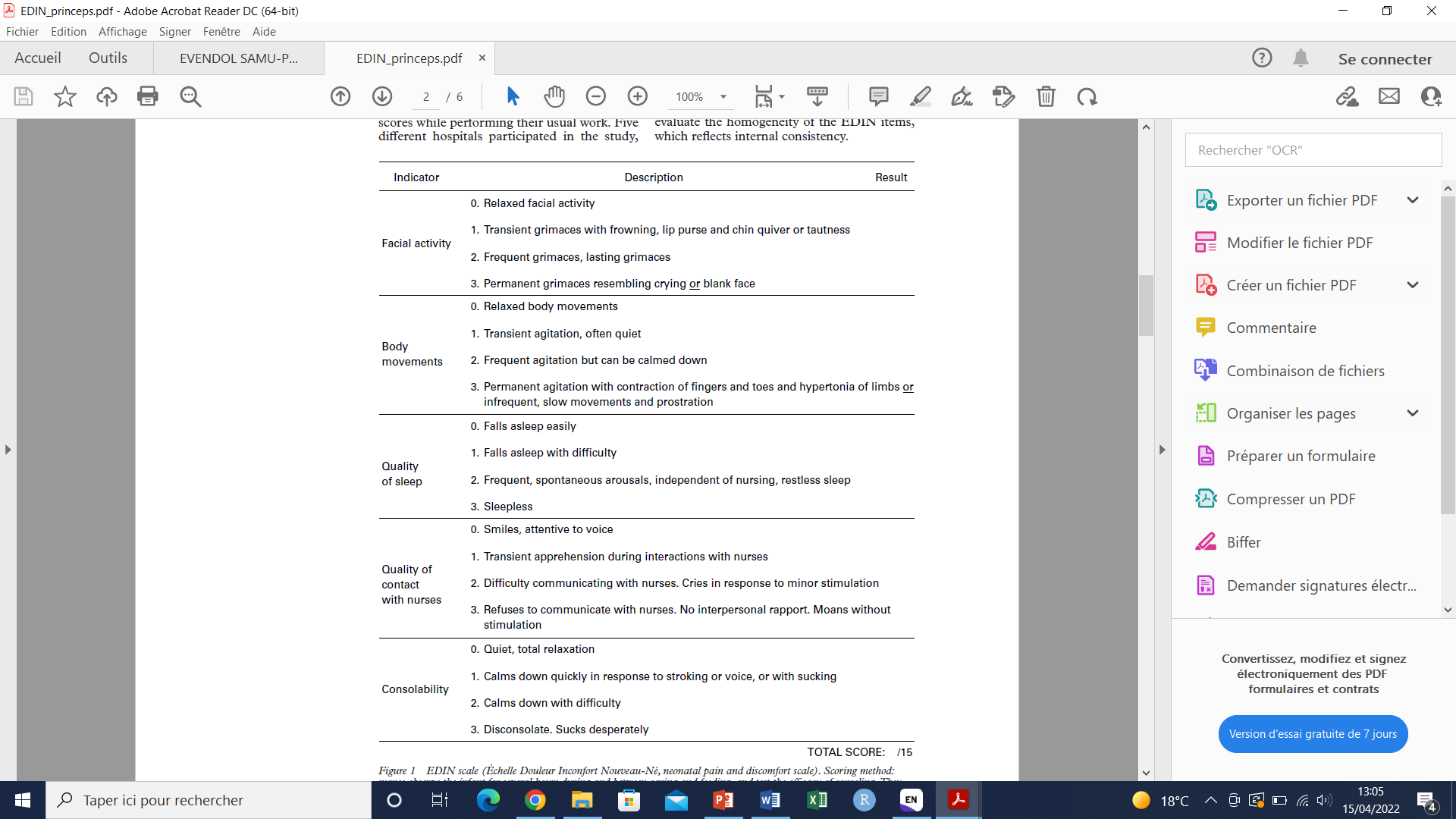


Supplemental Figure 1: the Echelle Douleur et Inconfort du Nouveau-né.


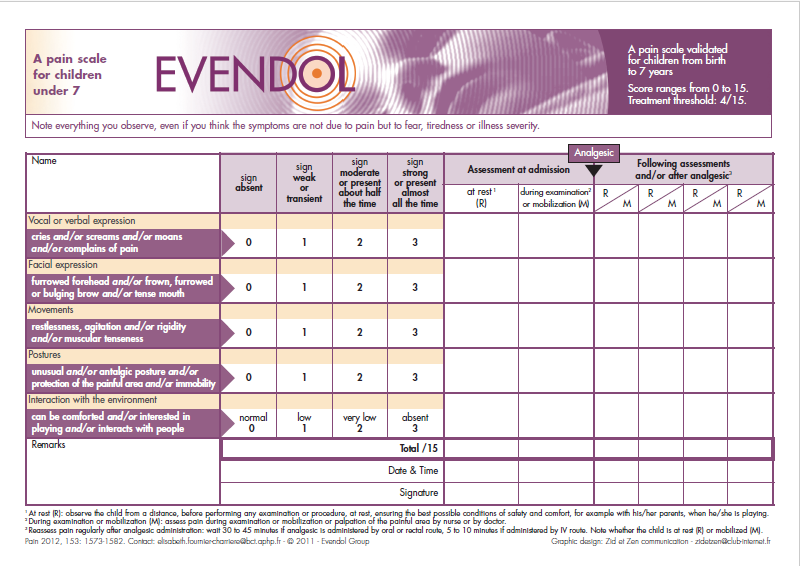


Supplemental Figure 2: the EVENDOL score.

Supplemental Table 3 : correlation between EVENDOL and the Numeric Rating Scale

between researcher and caregiver at rest and mobilisation

| **EVENDOL comparative** | **Spearman coefficient** | **p** |
| --- | --- | --- |
| **Rest** |  |  |
| NRS researcher | 0.78 | < 10^-3^ |
| NRS caregiver | 0.78 | < 10^-3^ |
| **Mobilisation** |  |  |
| NRS researcher | 0.93 | < 10^-3^ |
| NRS caregiver | 0.89 | < 10^-3^ |

NRS: Numeric Rating Scale
